# Social and Poor-Law Problems

**Published:** 1908-01-25

**Authors:** 


### SOCIAL AND POOR-LAW PROBLEMS.

### THE DECREASE OF NON-EMPLOYMENT.

During the year which ended March 31, 1907, 87,001 persons demanded help from the distress committees and the Central (Unemployed) Body for London, created under the Unemployed Act of 1905. Of this number 4,188 were women. Large as this number is, it is lower than the previous year, when the applications were 110,835. In some districts the conditions of labour were so good that either no action was taken—this was the case in Coventry, West Hartlepool, and Rhondda—or the committees received no applications—as happened in Cardiff, Gateshead, Huddersfield, St. Helen's, and Aston Manor. Taking the applications for relief all the country over, the proportion who asked for help was 5.7 per 1,000 of the populations. But the proportions varied largely in different districts. Thus in London as a whole the proportion of applications was only 6 per 1,000; but in Bermondsey, Poplar, and Woolwich, the proportion was 11 per 1,000; while in some parts of Greater London—for example, Edmonton, Erith, Leyton, Tottenham, Walthamstow, Croydon, and West and East Ham—the applications numbered 12 per 1,000.

Of the applications received, 14,832 were rejected for one reason or another. Some men had found occupation independently after making application for assistance, and others were rejected on account of their character. Still others were disqualified because they had already received Poor-law relief. The bulk of the applicants were men who were of full working age. Youths under twenty applied for help to the number of 2,318, and old men over sixty to the number of 3,025. Between fifty and sixty the number of applicants was 7,998. This leaves more than five-sixths of the whole applicants to have been persons between twenty and fifty. The actual division into age periods was, between twenty and thirty, 15,953; between thirty and forty, 17,171; and between forty and fifty, 13,930. As might be expected, the bulk of the applicants were casual labourers—either general labourers or connected with the building trades, where a good deal of unskilled labour is employed. The applicants who stated themselves to be general labourers numbered 31,534, and those connected with building 10,709. Next in number to these came the shipbuilding, engineering, and metal trades, who, combined, provided 4,107 applicants. Next to these came, strange to say, domestic servants, 1,659—a fact which indicates some economies on the part of the richer classes. From the boot and shoe trades came 1,429 applicants; from furnishing and wood working, 988; from the supplying of food, drink, and tobacco, 941; from the textile trades, 573; and from tailoring and clothing, 369; from the printing, bookbinding, and other paper trades, 308. The remaining 7,799 are put together under "other occupation," but it is probable that many of these might reasonably be classed as casual labourers. There are always a number of workers at season trades, both skilled and unskilled, who are apt to come on charity during the winter.

The number of applications actually dealt with amounted to 60,416. Of these persons 36,280 were provided with work. In some cases the local distress committees did not themselves provide work, but the local authority did, the distress committee contributing to the cost, either by bearing an agreed proportion of the expense, or by making up the additional cost which it was estimated was occurred through the exceptional character of the work and the limitations of the workers. A number of families were assisted to emigrate, for the most part to Canada or Australasia. The total number of persons thus assisted was 4,532, representing 1,112 men with 2,127 dependents from London, and 361 men with 932 dependents from the provinces. The cost of this emigration was £24,174 in London and £8,815 in the provinces. The distress committees have power also to assist unemployed persons to remove to other parts of the country, but this power was but little used. In all only 216 persons were thus sent to other parts of the country, of whom 117 were dependents. In all London only 47 persons were thus removed. The total cost of the operations of the committees in London and the provinces was £227,745; while the money received from all sources, including the subsidies from rates, Parliamentary grants, the Queen's Unemployed Fund, and voluntary charity, amounted to £244,844. Of this, £30,759 represented payment for work done. The largest item of expenditure was the payment for labour on farm and labour colonies, which amounted to £90,055.

The report is both interesting and helpful, and will become even more valuable when there are other annual reports to compare with it. For one thing which the public will want to know and cannot see from one report alone is whether or not the same persons come up winter after winter seeking aid. It is to be hoped that the local committees will note this point and record when the "regular out-of-work" presents himself. The intention of the Fund is to provide temporary help in a temporary emergency. If the unemployment threatens to become chronic or regularly periodic, the proper agency to deal with it is not a distress committee but the Poor-law.

